# p38 MAPKs — roles in skeletal muscle physiology, disease mechanisms, and as potential therapeutic targets

**DOI:** 10.1172/jci.insight.149915

**Published:** 2021-06-22

**Authors:** Christopher M. Brennan, Charles P. Emerson, Jane Owens, Nicolas Christoforou

**Affiliations:** 1 Rare Disease Research Unit, Pfizer Inc., Cambridge, Massachusetts, USA.; 2 Wellstone Muscular Dystrophy Program, Department of Neurology, University of Massachusetts Medical School, Worcester, Massachusetts, USA.

## Abstract

p38 MAPKs play a central role in orchestrating the cellular response to stress and inflammation and in the regulation of myogenesis. Potent inhibitors of p38 MAPKs have been pursued as potential therapies for several disease indications due to their antiinflammatory properties, although none have been approved to date. Here, we provide a brief overview of p38 MAPKs, including their role in regulating myogenesis and their association with disease progression. Finally, we discuss targeting p38 MAPKs as a therapeutic approach for treating facioscapulohumeral muscular dystrophy and other muscular dystrophies by addressing multiple pathological mechanisms in skeletal muscle.

## p38 family of MAPKs

**Figure 1:**
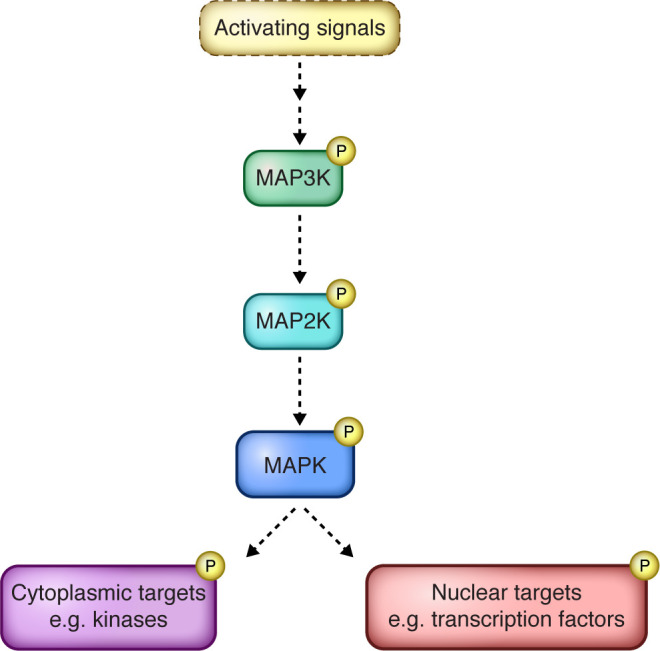
MAPK signaling cascade. Canonical mitogen MAPK signaling begins with an external activating signal, such as growth factors or stress, and proceeds through a series of activating phosphorylation events. MAPK kinase kinases (MAP3Ks) phosphorylate MAPK kinases (MAP2Ks), which phosphorylate MAPKs. Activated MAPKs facilitate the cellular response to the external signal by phosphorylating both cytoplasmic and nuclear proteins. These targets can include other central regulatory hubs, including other kinases and transcription factors. Illustrated by Rachel Davidowitz.

Mitogen-activated protein kinases (MAPKs) are serine/threonine kinases that are members of conserved signaling pathways that alter cellular physiology in response to extracellular signals (for review, see ref. 1). ERK, JNK, and p38 are major MAPKs in mammals, and like all MAPKs, are activated following a series of phosphorylation events. In response to external cues, MAPK kinase kinases (MAPKKKs, or MAP3Ks) phosphorylate MAPKKs (MAP2Ks) that then phosphorylate and activate MAPKs (Figure 1). MAPK signaling activity is regulated by cellular phosphatases that remove the activating phosphate groups. MAPK substrates include regulatory hubs, such as transcription factors and other signaling kinases, that control a variety of cellular processes, including cell division, inflammation, and differentiation.

The p38 MAPK family is composed of 4 related kinases, p38α (also known as MAPK14), p38β (MAPK11), p38γ (MAPK12), and p38δ (MAPK13; refs. 2, 3). p38α is ubiquitously expressed in human tissues, whereas expression of p38β and p38γ is largely restricted to brain and muscle, respectively (4). The 4 p38 family members are 60% conserved at the amino acid level and have largely overlapping substrates (3). Due to their similarity, many of the inhibitors and antibodies used in studies described in this Review are incapable of distinguishing between different p38 family members; however, distinctions are made here when possible. p38 MAPKs contain an N-terminal and C-terminal domain, with the ATP-binding site located at the junction of the 2 domains (5), and interact with substrates via docking sites in their C-terminal domain (6). These MAPKs are strongly activated by cytokines and cellular stress, which may arise due to osmotic, oxidative, UV light, and heat shock stress. These kinases can, however, be activated — albeit to a lesser extent and more transiently — by homeostatic functions, including myogenesis, as discussed in detail below. Three MAP2Ks (MKK3, MKK4, and MKK6) regulate p38 activity. MKK4 specifically regulates p38α and p38β; MKK6 is specific for p38γ and p38δ; and MKK3 phosphorylates p38α, p38β, and p38δ. Upstream of these MAP2Ks, several MAP3Ks regulate p38 signaling, and some respond to specific stresses. For example, ASK1 activates p38 in response to oxidative stress (7), while MEKK1 activates the pathway in response to UV radiation (8); thus, p38 kinases are able to integrate multiple external cues to control cellular functions.

Much of what has been learned about the substrates and function of p38 comes from studies with inhibitors of these kinases. Most p38 inhibitors bind within the ATP-binding pocket of the active site, competitively blocking ATP binding, thus inhibiting kinase activity (9). Due to the high degree of amino acid similarity in their active sites, many p38 inhibitors target both p38α and p38β (10). These inhibitors were first identified in phenotypic screens due to their ability to prevent cytokine production by LPS-stimulated human monocytes in vitro (11–13). Although several p38α/β inhibitors have been tested in clinical trials for a variety of inflammatory diseases (e.g., acute coronary syndrome, chronic obstructive pulmonary disease [COPD], and rheumatoid arthritis [RA]), none have yet gained regulatory approval due to lack of efficacy (14), as further discussed below.

p38α, p38β, and p38γ have well-established roles in skeletal myogenesis and muscle function, as recently reviewed by Segalés, et al. (15). Here, we explore the role of p38 in regulating skeletal muscle homeostasis, the disease process, and p38α/β targeting as an approach to treating muscular dystrophies, such as facioscapulohumeral muscular dystrophy (FSHD), that is currently undergoing clinical testing.

## Role of p38 kinases in skeletal myogenesis

The health of skeletal muscle depends on the maintenance of a robust population of quiescent muscle stem cells, called satellite cells, and their ability to become activated and differentiate to replace injured or diseased muscle. p38 kinases have multiple roles in this process, some of which are highlighted below. For in-depth review, see refs. 15 and 16.

### p38 in myogenic differentiation.

Myogenesis is the process by which activated satellite cells form proliferating myoblasts that differentiate and fuse to form multinucleated myofibers that constitute functional skeletal muscle. This process is controlled by basic helix-loop-helix (bHLH) transcription factors, namely Myf5, MyoD, myogenin, and MRF4, collectively referred to as myogenic regulatory factors (MRFs; ref. 17). Sequential expression and activation of MRFs promote progression of satellite cells through myogenesis. MyoD and Myf5 are expressed in uncommitted myoblasts; myogenin is expressed during early differentiation; and MRF4 is expressed during late differentiation (16, 18). MRFs dimerize with another class of bHLH transcription factors, Id (inhibitor of DNA binding) proteins, and with E proteins. Id proteins lack DNA-binding domains, and thereby inhibit transcription by MRFs (19). Downregulation of Id proteins at the onset of differentiation releases inhibition of MRFs, allowing for their activation (18). Heterodimers of MRFs and E proteins bind specific sequence elements (E boxes) within enhancers and promoters of genes involved in muscle differentiation and function (19). Additionally, proteins in the myogenic enhancer factor 2 (MEF2) family associate with MRFs and further enhance their transcriptional specificity and activity to express muscle protein–encoding genes (20).

**Figure 2:**
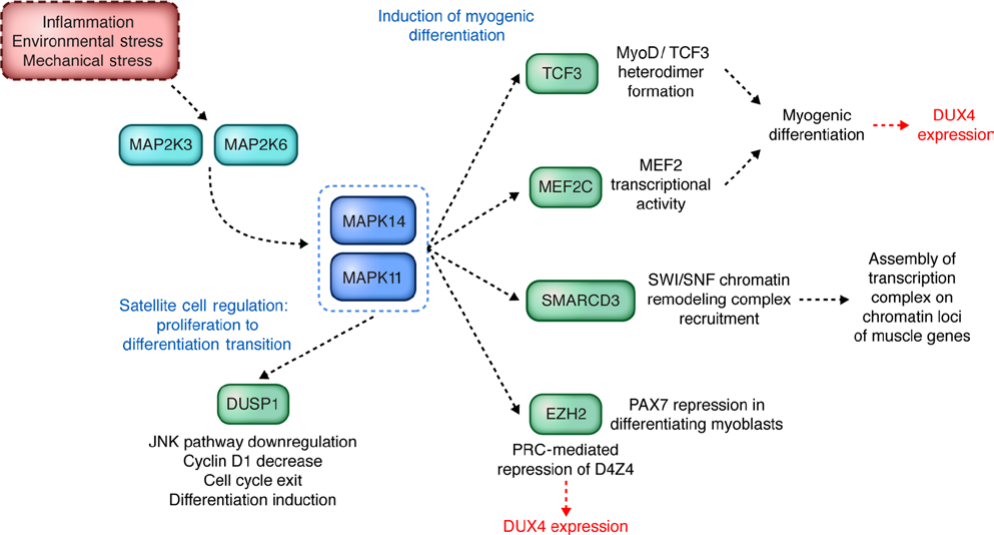
Roles of p38 MAPK in myogenic differentiation and satellite cell function for regulation of the FSHD disease gene *DUX4.* Members of the p38 family of MAP kinases are activated in response to stress but also to a lesser extent in muscle cells by the myogenic differentiation program. p38α (MAPK14) and p38β (MAPK11) can be activated by 2 different MAP2Ks, MAP2K3 (MKK3) and MAP2K6 (MKK6). p38α and p38β have both been shown to have roles in regulation of satellite cell fate and myogenic differentiation; however, the use of inhibitors that are potent against both p38α and p38β in many studies makes discerning their roles independent of one another difficult. Examples of the role of p38α/β in regulation of myogenesis are illustrated here and discussed in more detail in the main text. Potential points of DUX4 regulation by p38 are indicated in red. Illustrated by Rachel Davidowitz.

p38 is expressed and has an active role at the onset of differentiation. Multiple studies have reported its involvement in regulating MRF transcription of muscle protein genes during skeletal muscle differentiation (21–23) (Figure 2). First, p38α directly phosphorylates the MEF2 coactivator of MyoD to promote the formation of a functional MyoD-MEF2 heterodimer (24, 25) (Figure 2). Similarly, p38α/β phosphorylates E47 (also known as TCF3) to promote the formation of an active a MyoD-E47 heterodimer (26) (Figure 2). p38α also promotes myotube formation independently of MRFs by upregulating expression of the tetraspanin protein CD53, which localizes to the cell membrane and facilitates efficient myoblast fusion and myofiber formation (27). p38α also localizes to muscle gene regulatory elements bound by MRFs during differentiation, and p38α/β inhibitors prevent recruitment of the chromatin remodeling complex SWI/SNF to these loci (28). This recruitment is likely mediated through direct phosphorylation of BAF60C (also known as SMARCD3), a subunit of SWI/SNF reported to be involved in its interaction with transcription factors (28, 29) (Figure 2). Thus, p38α/β can promote transcription of myogenic genes by recruiting SWI/SNF to their regulatory elements, allowing the formation of euchromatin. Recent studies have shown that p38α binds to both active and inactive promoters at the onset of differentiation, further highlighting a role in transcription regulation, though whether or not p38 activity directly contributes to transcriptional repression has not been determined (30). p38α/β can also regulate the myogenic gene program posttranscriptionally via the RNA-binding protein KSRP, which binds RNA and promotes its degradation. During differentiation, KSRP is phosphorylated in a p38α/β-dependent manner, preventing it from binding and degrading myogenic transcripts. Thus, p38α/β effectively stabilizes myogenic transcripts (31). Additionally, there is evidence for crosstalk between signaling pathways activated by p38α/β and the MAPK family member JNK that promotes cell proliferation (Figure 1). Murine myoblasts derived from neonatal skeletal muscle genetically engineered to lack p38α fail to exit the cell cycle, even in differentiation-promoting conditions, and this coincides with an increase in JNK signaling and cyclin D levels. Importantly, this deficit can be rescued by pharmacological JNK inhibition, suggesting that increased JNK activity due to loss of p38α is specifically responsible for the inability of myoblasts to exit the cell cycle in order to begin differentiating (32). Mechanistically, p38α has been shown to inhibit the JNK pathway by upregulating the MAPK phosphatase MKP-1 (also known as DUSP1; ref. 32). Altogether, p38α/β positively regulates muscle cell differentiation on multiple levels and through many separate mechanisms.

Consequently, constitutive activation of p38 in myoblasts was shown to be sufficient to induce expression of differentiation markers and promote formation of multinucleated myotubes (23), whereas inhibition of p38α/β prevents induction of muscle-specific genes and myoblast fusion (21–23). In two studies, treatment of myoblasts with the p38α/β inhibitor SB203580 prevented formation of myotubes and expression of markers of both early and late myogenesis such as myogenin and creatine kinase, suggesting that p38α/β plays an important role early in myogenesis (21, 22). Another study found that the p38α/β inhibitor SB202190 blocked formation of myosin heavy chain–positive (MHC-positive) myotubes in multiple cell lines, including primary human myoblasts (23). In support of these studies, myoblasts derived from mice harboring genetic deletions in p38α also fail to undergo differentiation.

### p38 in satellite cell regulation.

Satellite cells are resident muscle stem cells that provide muscle with its ability to regenerate and repair (33). These cells reside beneath the basal lamina of the myofiber and remain in a quiescent state — controlled by expression of the transcription factor Pax7 — until activated by environmental cues, such as growth factors and cytokines, to proliferate as myoblasts (34, 35). Satellite cell activation leads to MyoD expression and subsequent reentry into the cell cycle (36–38). Like other stem cells, satellite cells can undergo asymmetric cell division such that one daughter is activated while the other remains quiescent, thereby renewing the stem cell pool (39). A small fraction of activated satellite cells retains stemness by downregulating MyoD, while the other fraction becomes committed progenitors by downregulating Pax7 (40). p38 provides a link between regenerative cues, MyoD induction, and Pax7 expression, thus controlling satellite cell activation and differentiation (Figure 2). Initially, p38α/β was shown to be expressed in activated satellite cells, and p38α/β inhibition caused satellite cells to exit the cell cycle and not undergo differentiation or respond to myogenic stimuli (41). This response was attributed to an inability of p38α/β activity–deficient cells to properly induce MyoD expression (41). Also, it was later discovered that p38 is critical for Pax7 repression through recruitment of polycomb repressive complex 2 (PRC2) to the *Pax7* promoter (42). TNF-α activates p38α, which then phosphorylates the PRC2 subunit EZH2. Phosphorylated PRC2 interacts with the transcription factor YY1, which binds upstream of the *Pax7* promoter to repress Pax7 expression, thus promoting cell cycle exit to quiescence (42). Interestingly, p38α/β also appears to be essential for asymmetric stem cell fate determination of activated satellite cells. p38α/β is activated in only one daughter cell, where it induces MyoD, thereby promoting division and differentiation. The other daughter cell, which lacks p38α/β, does not express MyoD and returns to a quiescent state (43). Thus, through the induction of MyoD and repression of Pax7, p38 promotes activation of quiescent satellite cells in response to myogenic stimuli.

p38γ regulates the decision for satellite cells to differentiate through phosphorylation of MyoD on Ser199 and Ser200, which promotes MyoD binding to the histone methyltransferase KMT1A. The MyoD-KMT1A repressive complex is recruited to the myogenin promoter, preventing expression and thereby blocking induction of the differentiation gene program (44). Consistent with this function, myoblasts from p38γ-deficient mice have decreased ability to fuse into multinucleated, MHC-positive myotubes (32).

## p38 promotes inflammation

p38α promotes inflammation by facilitating the production of proinflammatory cytokines, such as IL-1 and TNF-α (45), and can activate transcription factors, such as AP-1 family members, that induce cytokine expression (46, 47). In addition, p38α and p38β and their target MK2 promote cytokine expression by phosphorylating proteins that bind to AU-rich elements found in cytokine-encoding mRNA, thus stabilizing those transcripts (31, 48). Initial attempts to utilize p38α/β inhibitors therapeutically in the clinic focused on inflammatory conditions, such as rheumatoid arthritis (RA) (49, 50), neuropathic pain (51), focal segmental glomerulosclerosis (52), and COPD (14). In some cases, although p38α/β inhibitors were shown to be effective in preclinical models, they failed in clinical trials due to onset of unanticipated liver and neurological side effects (50, 53). Other inhibitors were shown to be safe at lower doses but conferred modest to no efficacy in humans (54, 55).

Inflammation also plays a major role in muscular dystrophy. Increased expression of cytokines and infiltration of immune cells are thought to be directly related to pathology (56, 57). For example, *IL17* mRNA is increased in muscle biopsies from patients with Duchenne muscular dystrophy (DMD) (58). In *mdx* mice, a mouse model of DMD, elevated IL-6 levels persist following injury compared with those in control mice and have been linked to muscle wasting (59). Moreover, steroid treatment to reduce inflammation is commonly used for DMD patients and effectively increases muscle strength while simultaneously delaying disease progression (60). p38α/β inhibition has the potential to decrease inflammation in muscular dystrophy, although its lack of success as a treatment for inflammatory diseases indicates that other aspects of muscular dystrophy pathology will need to be targeted in order for p38α/β inhibition to be an effective therapeutic. Below, we examine the potential of p38α/β inhibition in treating several diseases that affect the muscle, with a particular focus on FSHD.

## p38 in skeletal muscle disease

Most studies on p38 in muscle diseases to date have been conducted in preclinical models. For example, initial evidence for a role of p38 in DMD came from the finding that p38 is upregulated in exercise-trained *mdx* mice, but not in exercise-trained WT mice or in nonexercised *mdx* mice (61). The authors speculated that p38 has a role in degeneration of dystrophic muscle, as its upregulation was specific to exercise training, in which degeneration was exacerbated. Deletion of the dual-specificity phosphatase DUSP1, which normally acts to inhibit p38 activity, enhanced the dystrophic phenotype of *mdx* mice by preventing regeneration (62). Pharmacological inhibition of p38α/β improved the survival of *mdx* mouse myofibers challenged with oxidative stress, providing evidence that p38α/β inhibition could be used as a potential therapy for DMD (63). A comprehensive study by Wissing et al. found that p38α deficiency in the *mdx* mouse model and the sarcoglycan-knockout mouse (*Sgcd^–/–^*), a model for limb-girdle muscular dystrophy, alleviated pathology in both models, decreasing fibrosis and macrophage infiltration and improving endurance on a treadmill (64). p38 activation also appeared to be at least partially sufficient for development of some muscular dystrophy phenotypes, as artificially increasing p38 activity by expression of constitutively active MKK6 induced muscle degeneration pathology reminiscent of that observed in muscular dystrophies. It is worth noting that MKK6 expression likely increased p38 activity beyond physiological levels and could thus cause toxicity. The authors went on to demonstrate that these effects were due to p38 phosphorylating and activating the proapoptotic factor Bax (64). Taken together, these data suggest that p38 inhibition may be beneficial for treatment of multiple forms of muscular dystrophy by preventing inflammation and myofiber death.

## p38 inhibitors as a therapy for LMNA-related heart disease

Inhibition of p38α is currently being pursued as a potential treatment for LMNA-related dilated cardiomyopathy (DCM) (ClinicalTrials.gov, NCT02057341). This form of heart disease is caused by mutations in *LMNA*, which encodes nuclear A/C type lamins (65). Mutations in *LMNA* lead to biomechanical defects in cardiomyocytes due to loss of nuclear integrity, ultimately causing cardiac dilation and progressive heart failure (66). In contrast to the previously described studies where beneficial effects of p38α/β inhibition were due to prevention of p38-mediated inflammation and apoptosis, p38 signaling has been shown to be proximal to the mutation that causes LMNA-related DCM. Analysis of the transcriptomes of heart tissue from a LMNA mutation–harboring mouse model pointed to hyperactivation of the p38 pathway prior to the onset of cardiac disease phenotypes (67). Treatment with a p38α inhibitor prevented left-ventricle dilation and ameliorated cardiac phenotypes caused by LMNA mutations. The mechanism for this would later be proposed to be the mechanical correction of cytoskeletal defects upon p38α inhibition (68). Three separate LMNA mutations were shown to cause mechanical defects in cardiomyocytes, including reduced cytoskeletal elasticity and adhesion, by use of atomic force microscopy (AFM). These biomechanical defects were rescued by treatment with a p38α inhibitor (68). The authors proposed that p38 may be contributing to the altered biomechanical features of LMNA mutant cardiomyocytes via its established role in regulating the actin cytoskeleton, and thus these p38-mediated effects may have led to the observed loss of contractility and increased apoptosis (69–71). Indeed, cardiomyocytes harboring mutations in LMNA have a disorganized network of actin filaments, and treatment with a p38α inhibitor restored them to a WT-like state (68). Thus, rather than preventing inflammation and apoptosis, p38α inhibition may correct some of the mechanical flaws caused by defective lamin in cardiac cells due to genetic mutations. It is worth noting that patients with LMNA mutations can also present with varying degrees of skeletal myopathy, including Emery-Dreifuss muscular dystrophy and limb-girdle muscular dystrophy (72–74). It would be interesting to explore the potential of p38α inhibitors to improve skeletal muscle phenotypes associated with LMNA mutations as well.

## p38 inhibitors as a multitargeted therapy for FSHD

Inhibition of p38α/β is also being pursued as a potential treatment for FSHD (for review, see ref. 75). FSHD is a progressive muscle degenerative disease that affects approximately 1:15,000 individuals (76). This dystrophy is typically diagnosed in the second or third decade of life, though a more-severe infantile form exists. Initially, muscles of the face, shoulder, and upper arms are affected; and as the disease progresses, it causes degeneration of muscles in the lower extremities, as well as loss of mobility and inability to perform daily tasks (75). There are 2 genetic forms of FSHD. FSHD1 is more common and is caused by a contraction in the number of D4Z4 macrosatellite repeats in the subtelomeric region of the q arm of chromosome 4 (77). FSHD2 is caused by mutations in epigenetic regulators, most commonly the chromatin modifier SMCHD1 (78). Both mutations lead to inappropriate expression of DUX4, a transcription factor encoded within the D4Z4 repeats that is typically only expressed during early embryonic development, due to epigenetic de-repression of the D4Z4 region (79, 80). In cultured myoblasts isolated from FSHD patient biopsy specimens, only a small fraction of myonuclei express DUX4 following induced differentiation into myotubes (80). DUX4 expression in myonuclei leads to aberrant expression of many transcripts that are typically restricted to the cleavage-stage embryo (81–84). Additionally, DUX4 interacts with critical RNA-binding proteins involved in RNA splicing and translation, potentially altering their function (85). It is not yet understood whether DUX4 toxicity is due to misexpression of its transcriptional targets or dominant interactions with essential cellular proteins. However, the net result of DUX4 misexpression in FSHD myotubes or cells engineered to conditionally express DUX4 is induction of many cellular stresses — including oxidative stress (86–88), proteotoxic stress (89), and accumulation of double-stranded RNA (90) — that ultimately lead to the death of the myofiber. Precisely how DUX4 kills cells is also an area of active investigation. Multiple studies have found that DUX4 expression activates caspase-3/7 via a p53-dependent mechanism (91–93), but p53-independent cell death due to DUX4 expression has also been described (90, 94).

Pathological features of biopsy samples from affected muscle tissue of FSHD patients include degenerating and regenerating fibers; replacement of muscle with fatty-fibrotic tissue; and pronounced inflammation, particularly immune cell infiltration (95). Analysis of the transcriptome of affected muscle tissue in patients with FSHD revealed downregulation of genes involved in muscle development and differentiation, and upregulation of extracellular matrix, inflammatory, and immune response genes (81, 96). Thus, DUX4 expression causes FSHD-associated symptoms by inducing muscle fiber degeneration and promoting inflammation. How the immune system responds to FSHD muscles and contributes to pathology is an area of active investigation.

### Inhibition of p38α/β decreases expression of DUX4, the FSHD disease gene.

Interestingly, a screen for molecules that prevent DUX4 expression in FSHD patient–derived muscle cells uncovered multiple p38α/β inhibitors as potent repressors of the DUX4 target gene *MBD3L2* (97). Further examination of the effects of these compounds on FSHD patient–derived muscle cells led to the discovery that p38 inhibition causes a reduction in DUX4 mRNA that in turn results in decreased mRNA levels of several DUX4 target genes (97, 98). Importantly, p38α/β inhibition decreased cell death in FSHD patient cells (97). As DUX4 is a primate-specific gene, animal models of FSHD are lacking. However, p38α/β inhibitor treatment of humanized mice with FSHD patient–derived muscle xenografts reduced DUX4 expression, target gene expression, and apoptosis in xenografted muscle (98). Notably, FSHD xenografts are generated in immune-deficient mice and thus fail to model the role of immune cell–mediated inflammation, which may also provide a treatment mode for p38α/β inhibitors. Thus, p38α/β inhibition in preclinical models of FSHD appears to be targeting the root cause of the disease by decreasing DUX4 expression. These data provided the basis for clinical development of p38 inhibitors for treating FSHD, which is discussed below.

Currently, the mechanism by which p38 controls DUX4 expression in muscle is not fully understood. DUX4 is not expressed in proliferating FSHD patient myoblasts in cell culture, and its expression is increased in only a small fraction of myotube nuclei following myotube differentiation (80, 83). p38α/β inhibitors block DUX4 mRNA expression as well as disrupting MyoD/MRF transcription factor function, as described above, supporting a role for p38 in modulating DUX4 expression as part of the differentiation transcriptional regulatory response (Figure 2). It is worth noting that treatments with concentrations of p38α/β inhibitors up to 1 μM still inhibited DUX4 expression but had no effect on the formation of myotubes or expression of differentiation markers (97, 98). However, earlier studies showing p38α/β inhibition of myogenesis used inhibitor concentrations that were between 5- and 20-fold higher, indicating that inhibition of differentiation and DUX4 expression is concentration dependent (21–23). Furthermore, RNA sequencing (RNA-Seq) analysis of FSHD myotubes treated with 2 different p38α/β inhibitors revealed that the majority of statistically significant changes in gene expression consisted of reduced levels of DUX4 target genes, not myogenic transcripts (97). In this experiment, only a single time point later in differentiation was analyzed, leaving the possibility that p38α/β inhibition causes delays in myogenic differentiation. A recent CRISPR genome screen for DUX4 toxicity reported that DUX4 function is also controlled by hypoxia signaling (99). DUX4 expression itself leads to induction of hypoxia, as measured by stabilization of HIF-1, the hypoxic response master regulator. Interestingly, pharmacological inhibition of hypoxia signaling decreased DUX4 protein stability, DUX4 expression targets, and apoptosis. Several studies have demonstrated that p38 regulates hypoxia signaling (100–102), so perhaps this is one mechanism by which p38α/β inhibition decreases DUX4-dependent gene expression. Alternatively, p38 could regulate transcription factors or chromatin modifiers that control the epigenetic status of the D4Z4 repeat locus in which DUX4 is expressed from the terminal repeat. For example, the polycomb repressive complex (PRC) epigenetically represses the D4Z4 repeat by maintaining repressive H3K27me3 marks, and the PRC subunit EZH2 is a known target of p38α in satellite cells (103, 104) (Figure 2). It will be interesting to determine whether other DUX4-repressing proteins are targets of p38α/β inhibitors in muscle to further understand whether p38 controls DUX4 expression primarily through epigenetic repressors.

### Additional potential therapeutic effects of p38α/β inhibition in FSHD.

p38 plays a critical role in controlling myogenesis (highlighted above); therefore, any potential side effects of pharmacological p38α/β inhibition that affect satellite cell regulation or myogenic differentiation are of concern. In particular, chronic inhibition of p38α/β has the potential to result in a reduction in the ability of satellite cells to proliferate as myoblasts and further differentiate to repair and replace damaged myofibers. The effects of long-term treatment with p38 inhibitors on muscle regeneration have yet to be fully explored. As muscle in patients with FSHD shows evidence of active regeneration based on expression of embryonic isoforms of myosin and the presence of centrally located nuclei, chronic p38α/β inhibition as a long-term treatment may not be feasible (105). As noted above, at low concentrations, these inhibitors did not affect early stages of myogenesis in FSHD xenograft muscle studies (97, 98). Monitoring the effects of inhibitors on muscle physiology and regeneration during clinical testing of p38α/β inhibitors in FSHD will be important for evaluating the expected long-term treatment plan needed for therapeutically effective use of these inhibitors, as previous trials with p38α/β inhibitors for other nonmuscle indications did not specifically examine muscle toxicology.

Similar to other muscular dystrophies where p38α/β inhibition has been tested in preclinical models (64), FSHD is associated with significant inflammation and a distinct innate and adaptive immune signature (106). Therefore, p38α/β inhibitors may provide additional benefits for patients with FSHD independent of DUX4 repression by targeting and preventing inflammation and Bax-dependent apoptosis, as has been observed in *mdx* and *Sgcd*^–/–^ mouse models of muscular dystrophy (64). Preclinical models of FSHD inflammation that would enable this hypothesis to be tested are currently under development and will be valuable tools for future FSHD clinical trial development. It is also now possible to gain information on the infiltrating immune cell types in FSHD muscle during disease progression using high-spatial-resolution RNA-Seq and antibody protein mapping of muscle (107). These methods could be of value for analyzing the effects of therapies on inflammation in FSHD clinical trials. Additionally, p38α/β inhibitors are known to prevent p53-dependent apoptosis. Mechanistic studies have shown that p38 directly phosphorylates p53 in response to viral infection and DNA damage caused by either UV radiation or chemotherapy, thereby promoting p53-dependent transcription and apoptosis (108–110), another indication that p38α/β is a favorable target for inhibition in FSHD therapy (91–93).

### Losmapimod clinical trial and a future outlook.

Among several p38α/β inhibitors demonstrated to be efficacious in blocking DUX4 functions in cells from patients with FSHD, the p38α/β inhibitor losmapimod was selected for clinical trials in part due to its positive clinical safety profile in humans. In a trial for COPD, losmapimod was well tolerated in more than 3500 participants (54, 55) but failed to demonstrate efficacy. More recently, a phase I study evaluated losmapimod in FSHD patients. There was a dose-dependent increase in losmapimod exposure in muscle, as well as evidence of target engagement, as measured by levels of phosphorylated Hsp27, an indirect target and surrogate marker of p38 function. Losmapimod accumulated to a concentration of approximately 100 nM in muscle, with a dosing regimen of 15 mg per day (111), a level that was sufficient to inhibit DUX4 function in patient cell assays (97). The ReDUX4 phase II trial (ClinicalTrials.gov, NCT04003974) is currently ongoing, with results expected to be presented at the FSHD Society International Research Congress, June 24–25, 2021. The primary end point for this study is robust downregulation of DUX4-activated gene expression as measured by RNA levels of several DUX4 target genes in MRI-guided muscle biopsies collected after 16 and 36 weeks of treatment. Clinical measurements of mobility and muscle strength will also be made. Interim data showed little difference in DUX4-activated gene expression between placebo-treated and losmapimod-treated groups when looking at all trial participants; however, a decrease in DUX4-activated gene expression was observed in losmapimod-treated participants with the highest baseline levels of DUX4-activated gene expression (112).

The lack of relevant animal models to test the action of losmapimod on the multiple p38 targets, apart from the human xenograft model for DUX4, limits development of clinical trial protocols, but such information can be generated as part of future p38α/β inhibitor trials. It remains to be determined what levels of DUX4 inhibition are required for effective clinical outcomes, and whether low levels of DUX4 expression are sufficient or if expression must be entirely eliminated to achieve a reasonable outcome for improvement of patient quality of life. It also remains to be determined whether p38α/β inhibitor treatment will significantly reverse muscle damage or merely prevent further disease progression. The results of the ongoing losmapimod trial will provide an indication of the potential of p38α/β inhibitors as therapeutics for FSHD. Moreover, the results will provide an invaluable molecular and clinical platform to enable future FSHD clinical trials, offering hope for treatment of this devastating disease.

## Version 1 06/22/2021

Electronic publication
